# A single-center experience with pancreatic cystic neuroendocrine tumors

**DOI:** 10.1186/s12957-020-01994-6

**Published:** 2020-08-15

**Authors:** Ange Khalil, Jacques Ewald, Ugo Marchese, Aurélie Autret, Jonathan Garnier, Patricia Niccoli, Gilles Piana, Flora Poizat, Marc Giovannini, Jean-Robert Delpero, Olivier Turrini

**Affiliations:** 1 Department of Surgery, ENETS co-E IPC NET Center, Institut Paoli-Calmettes, 232 Boulevard Sainte Marguerite, 13009 Marseille, France; 2 Department of Biostatistics, Institut Paoli-Calmettes, Marseille, France; 3 Department of Oncology, Institut Paoli-Calmettes, Marseille, France; 4 Department of Radiology, Institut Paoli-Calmettes, Marseille, France; 5 Department of Pathology, Institut Paoli-Calmettes, Marseille, France; 6 Department of Endoscopy, Institut Paoli-Calmettes, Marseille, France; 7 Department of Surgery, CNRS, Inserm, CRCM, Institut Paoli-Calmettes, Aix-Marseille University, Marseille, France

**Keywords:** Pancreatic neuroendocrine tumor, Survival, Cystic component

## Abstract

**Background:**

Pancreatic neuroendocrine tumors (PNET) are rare, with a significant malignant potential. This study aimed to determine outcomes of patients with resected PNETs according to the cystic component and confirm the accuracy of preoperative staging.

**Methods:**

From 1997 to 2016, 106 patients underwent resection of PNETs, including 73 purely solid (S-PNETs, 69%), 21 mixed (M-PNETs, 20%), and 12 purely cystic lesions (C-PNETs, 11%). To ensure consistent comparisons of overall (OS) and disease-free (DFS) survival outcomes between the 3 groups, the patients were matched according to the World Health Organization (WHO) grade and tumor height.

**Results:**

Overall, the rate of correlation between the preoperative and pathological diagnoses was low in the C-PNET group (33%, *P* = 0.03). None of the 24 patients (23%) with metastatic disease at the time of surgery were in the C-PNET group. Furthermore, significantly more parenchyma-sparing resections (*P* = 0.039) and fewer enlarged resections (*P* = 0.019) were achieved in the C-PNET group. C-PNET group had a significantly lower node invasion rate than the S-PNET and M-PNET groups (8% vs. 41% and 24%, *P* = 0.004). Although median OS was comparable in all 3 groups before (*P* = 0.3) and after (*P* = 0.18) matching, higher median DFS was observed in the C-PNET group than in the other groups after matching (*P* = 0.038).

**Conclusion:**

C-PNET was associated with a better prognosis than PNET with a solid component. The results support a wait-and-see policy in cases wherein a reliable preoperative diagnosis remains challenging.

## Introduction

Pancreatic neuroendocrine tumors (PNET) are a rare disease with a significant malignant potential but with a better overall survival (OS) prognosis than that associated with adenocarcinoma or cholangiocarcinoma [1–3]. The steadily increasing incidence of PNET over the last 20 years has been attributed mainly to increased incidental detection during the performance of non-specific imaging [computed tomography (CT), magnetic resonance imaging (MRI), endoscopy ultrasound (EUS)].

A PNET may appear as a purely solid (S-PNET), purely cystic (C-PNET), or solid-cystic tumor (mixed, M-PNET) on a radiologic image. C-PNETs, which account for 10% of all PNETs [2, 3], are considered more indolent with a correspondingly more favorable prognosis when compared with other PNETs. These favorable characteristics have led to conflicting opinions regarding the usefulness of therapeutic C-PNET resection. However, the diagnosis of C-PNET remains challenging because the misdiagnosis of a unique cystic pancreatic tumor could lead either to a failure to resect a potentially malignant cystic lesion [e.g., mucinous cystadenoma, intrapapillary mucinous tumor (IPMN)] or to the performance of an unnecessary surgery associated with high morbidity for a benign lesion (e.g., serous cystadenoma). Consequently, several studies have evaluated lesion biopsies and/or cyst fluid with the aim of increasing the accuracy of C-PNET. However, the results have been varied and disappointing.

Despite the continued uncertainty, all solid and cystic pancreatic tumors are staged according to preoperative imaging and biopsy findings and/or cyst fluid analysis results. Therefore, our study had two objectives. First, we aimed to determine the outcomes of all resected PNETs according to the cystic component. Second, we aimed to determine the accuracy of preoperative staging with respect to a C-PNET diagnosis.

## Materials and methods

From January 1, 2000, to December 31, 2016, 1096 patients underwent pancreatectomy surgery at the Paoli-Calmettes Institute, Marseille, France. All patients were included in a registered and prospectively maintained database (CHIRPAN No. Sy50955016U). Patients who (a) underwent pancreatectomy for non-PNETs or mixed PNET/adenocarcinoma, (b) had an unclear pathologic analysis, or (c) had incomplete follow-up data were excluded from the present study. Finally, 106 patients comprised our population study.

### Preoperative staging

All patients were initially staged based on findings from a physical examination and imaging as thoraco-abdominal CT scan/MRI, EUS with fine-needle aspiration of the solid and/or cystic component, ^68^Ga-DOTATATE PET/CT, and ^99m^Tc-Octreotide SPECT/CT. After staging and before any treatment, all cases were discussed by a multidisciplinary tumor board. PNET diagnosis was established regarding on all data collected. Patients were re-staged within 4 weeks preoperatively if a neoadjuvant treatment was administered.

### Surgery

Resection surgeries were performed by 3 experienced pancreatic surgeons according to the tumor location and staging. Enlarged resection and portal vein/superior mesenteric vein resection were performed as needed. Synchronous resection (e.g., pancreas and liver) was performed in cases involving hepatic metastasis. Drainage was achieved according to the procedure type and surgeon’s choice. Postoperative octreotide was not routinely administered to prevent postoperative pancreatic fistula (POPF). Adjuvant treatment was administered according to the decision of the multidisciplinary tumor board after a pathologic analysis.

### Pathological analysis

A purely cystic tumor (C-PNET) was defined by the complete absence of a solid component. A purely solid tumor (S-PNET) was defined by the total absence of a cystic component. A mixed tumor (M-PNET) was defined by the presence of contiguous cystic and solid components. MIB and/or Ki67 were used interchangeably by pathologists as markers for differentiation grading. Ki67 was used as surrogate of differentiation: a low Ki67 corresponded to a highly differentiated tumor and conversely a low Ki67 resulted in a low-grade tumor.

### Follow-up

The follow-up was conducted by a clinical exam and CT-scan every 4 months by a surgeon and/or an oncologist, with a minimum follow-up of 1 year. Patients lost to follow-up are those for whom there has been no news for 1 year. Specific and non-specific deaths were noted. Overall survival was calculated from the operative date until the date of the latest news (lost to follow-up or death). Disease-free survival was calculated from the date of the surgery to the date of diagnosis of the recurrence. The recurrence was defined by the appearance of a locoregional recurrence or a metastatic disease or the evolution of known metastases.

### Study parameters

The age, sex, body mass index, symptoms, metastasis, perioperative treatments (e.g., chemotherapy and/or octreotide), tumor markers (chromogranin A and gastrin), multiple endocrine neoplasia type 1, or Von–Hippel–Lindau diseases, supposed diagnosis after initial staging, and tumor location (head, body, or tail) were recorded for each patient. Additionally, the surgical approach (laparotomy, laparoscopy, or robot-assisted), parenchyma-sparing surgery (enucleation or central pancreatectomy) or not (pancreaticoduodenectomy, left pancreatectomy with or without splenectomy, or total pancreatectomy), resection extent (e.g., vascular resection, adjacent organ resection, liver metastasis resection), pancreas texture, and operative duration (minutes) were also recorded. The postoperative complications (including POPF) reported according to the Clavien–Dindo classification (mortality was determined within 30 postoperative days or before patient discharge), hospital stay length (days), and readmissions were also noted. Pancreatic insufficiency was defined as the diagnosis of steatorrhea and/or diabetes within the first 6 months after surgery. The tumor height (mm), PNET type (S-PNET, C-PNET, or M-PNET), margin resection status (R0, R1, or R2), number of resected lymph node, lymph node invasion, metastasis status, vascular invasion, and MIB and/or Ki67 index were also recorded. Regarding outcomes, recurrence events (date and location) and patient deaths (date and cause) were recorded. Finally, each PNET was classified according to the 2017 World Health Organization (WHO) and TNM classifications.

### Statistical analysis

Data analyses were performed using the GraphPad Prism software, version 5.0d (GraphPad Software Inc., La Jolla, CA, USA) and the SAS statistical software version 9.1 (SAS Institute, Inc., Cary, NC, USA). Qualitative variables were summarized by frequencies and percentages; quantitative variables were summarized using position and variability statistics as median and range. Differences between the groups were assessed using the chi-squared test for categorical variables and the Wilcoxon rank-sum test for continuous variables. Probabilities of OS and DFS were calculated using the Kaplan-Meier method and comparisons were evaluated with log-rank test. The level of statistical significance was set at a *P* value < 0.05.

We additionally applied a 1:5 matching procedure based on the WHO grade, tumor height, BMI, and age to ensure a consistent comparison between patients of all 3 groups. The matching was made with the macro-match available on the website of Duke University (supplementary Table 4).

## Results

The characteristics of the 106 patients included in our analysis are summarized in Table 1. According to the pathological analysis, 73 (68.9%), 21 (19.8%), and 12 (11.3%) patients were diagnosed with S-PNET, M-PNET, and C-PNET, respectively. A total of 24 patients (22.6%) had metastatic disease, and the incidence of metastasis was significantly more frequent in the S-PNET group relative to the C-PNET group (*P* = 0.027).

**Table 1:** Population characteristics

**Variables**	**All (*n*** **= 106)**	**C-PNETs** **(*n*** **= 12)**	**M-PNETs (*n*** **= 21)**	**S-PNETs (*n*** **= 73)**	***P*** **value (C Vs S)**	***P*** **value (C Vs M)**	***P*** **value (M Vs S)**
**Sexe, female (%)**	64 (60.4)	7 (58.3)	14 (66.7)	43 (58.9)	Ns	Ns	Ns
**Weight, median [min-max]**	67 [10-99]	74 [45-95]	71.50 [45-85]	64.5 [10-99]	Ns	Ns	Ns
**Size (m), median [min-max]**	1.7 [1.4-1.9]	1.68 [1.5-1.8]	1.7 [1.5-1.8]	1.7 [1.4-1.9]	Ns	Ns	Ns
**BMI > 25%**	36 (37.9)	8 (66.7)	9 (42.9)	19 (26)	**0.005**	Ns	Ns
**Age at surgery > 60 years old (%)**	47 (44.3)	3 (25)	10 (47.6)	34 (46.6)	Ns	Ns	Ns
**Symptoms (%)**	75 (70.8)	7 (58.3)	16 (76.2)	52 (71.2)	Ns	Ns	Ns
**Functional (%)**	11 (10.4)	0	2 (9.5)	9 (12.3)	Ns	Ns	Ns
**MEN 1 (%)**	8 (7.5)	2 (16.7)	2 (9.5)	4 (5.5)	Ns	Ns	Ns
**VHL (%)**	5 (4.7)	0	0	5 (6.8)	Ns	Ns	Ns
**Biopsy (%)**	96 (90.6)	11 (91.7)	18 (85.7)	67 (91.8)	Ns	Ns	Ns
**Imaging**							
Thoraco-abdominal CT-Scan (%)	104 (98.1)	12 (100)	20 (95.2)	72 (98.6)	Ns	Ns	Ns
EUS with fine needle aspiration (%)	95 (89.6)	12 (100)	18 (85.7)	65 (89.0)	Ns	Ns	Ns
Abdominal MRI (%)	57 (53.8)	6 (50)	15 (71.4)	36 (49.3)	Ns	Ns	Ns
^68^Ga-DOTATATE PET/CT (%)	8 (7.6)	0 (0)	1 (4.8)	7 (9.6)	Ns	Ns	Ns
^99m^Tc-Octreotide SPECT/CT (%)	37 (34.9)	4 (33.3)	7 (33.3)	26 (35.6)	Ns	Ns	Ns
**Preoperative diagnosis of NET (%)**	82 (77.4)	4 (33.3)	13 (61.9)	65 (89)	**< 0.001**	Ns	**0.004**
**Metastasis at staging (%)**	24 (22.6)	0	2 (9.5)	22 (30.1)	**0.027**	Ns	Ns
**Neo-adjuvent chemotherapy (%)**	8 (7.5)	0	0	8 (11)	Ns	Ns	Ns
**Adjuvent chemotherapy (%)**	23 (21.7)	0	3 (14.3)	20 (27.4)	**0.038**	Ns	Ns
**Neo-adjuvent Sandostatin analog (%)**	6 (5.7)	0	1 (4.8)	5 (6.8)	Ns	Ns	Ns

Abbreviations: *BMI* body mass index, *MEN 1* multiple endocrine neoplasia type 1, *VHL* Von-Hippel-Lindau syndrome, *CT-Scan* computerized tomography scan, *EUS* endoscopic ultrasonography, *MRI* magnetic resonance imaging, *PET/CT* positron emission tomography scan; *SPECT/CT* single photon emission computed tomography; *NET* neuro-endocrine tumor

### Surgery

Details of the surgeries and postoperative courses are summarized in Table 2. Patients with C-PNETs underwent significantly more parenchyma-sparing resections (*P* = 0.039) and significantly fewer enlarged resections (*P* = 0.019), compared with those in the other groups. The overall mortality and morbidity rates were 3.8% and 54.7%, respectively, and these did not differ significantly between the 3 groups. The C-PNET group had a significantly shorter operative duration (249.1 vs 419.6 min, *P* = 0.001) and hospital stay length (12.8 vs 17.6 days, *P* = 0.007), higher readmission rate (25% vs 5.5%, *P* = 0.023), and lower pancreatic insufficiency rate when compared with the S-PNET group (0% vs 27.4%, *P* = 0.038).

**Table 2:** Surgery and postoperative courses

**Variables**	**All (*n*** **= 106)**	**C-PNETs (*n*** **= 12)**	**M-PNETs (*n*** **= 21)**	**S-PNETs (*n*** **= 73)**	***P*** **value (C Vs S)**	***P*** **value (C Vs M)**	***P*** **value (M Vs S)**
**Approach**							
Laparotomy (%)	89 (84)	8 (66.7)	19 (90.5)	62 (84.9)	Ns	Ns	Ns
Laparoscopy (%)	14 (13.2)	4 (33.3)	1 (4.8)	9 (12.3)	Ns	**0.028**	Ns
Robot-assisted (%)	3 (2.8)	0	1 (4.8)	2 (2.7)	Ns	Ns	Ns
**Type of surgery**							
PD (%)	30 (28.3)	1 (8.3)	4 (19)	25 (34.2)	Ns	Ns	Ns
DP (%)	54 (50.9)	8 (66.7)	9 (42.9)	37 (50.7)	Ns	Ns	Ns
Central pancreatectomy (%)	6 (5.7)	1 (8.3)	2 (9.5)	3 (4.1)	Ns	Ns	Ns
Enucleation (%)	15 (14.2)	2 (16.7)	6 (28.6)	7 (9.6)	Ns	Ns	**0.026**
Completion of pancreatectomy (%)	2 (1.9)	0	1 (4.8)	1 (1.4)	Ns	Ns	Ns
Enlarged resection (%)	32 (30.2)	0	8 (38.1)	24 (32.9)	**0.019**	**0.014**	Ns
Parenchyma sparing (%)	20 (18.9)	3 (25)	7 (33.3)	10 (13.7)	Ns	Ns	**0.039**
**Morbidity** (Clavien-Dindo grading system)							
I (%)	10 (9.4)	0	3 (14.3)	7 (9.6)	Ns	Ns	Ns
II (%)	21 (19.8)	1 (8.3)	5 (23.8)	15 (20.6)	Ns	Ns	Ns
III (%)	18 (17)	4 (33.3)	6 (28.6)	8 (11)	Ns	Ns	Ns
IV (%)	5 (4.7)	1 (8.3)	0	4 (5.5)	Ns	Ns	Ns
V (%)	4 (3.8)	0	1 (4.8)	3 (4.1)	Ns	Ns	Ns
**Type of complication**							
Pancreatic fistula (%)	40 (37.7)	5 (41.7)	12 (57.1)	23 (31.5)	Ns	Ns	**0.032**
Hemorrhage (%)	8 (7.5)	1 (8.3)	0	7 (9.6)	Ns	Ns	Ns
Diabetes (%)	22 (20.8)	2 (16.7)	6 (28.6)	14 (19.2)	Ns	Ns	Ns
EPI (%)	25 (23.6)	0	5 (23.8)	20 (27.4)	**0.038**	Ns	Ns
**Interventional drainage (%)**	20 (18.9)	5 (41.7)	6 (28.6)	9 (12.3)	**0.011**	Ns	Ns
**Operating time (min), median [min-max]**	372 [120-960]	230 [120-471]	412 [205-725]	387.5 [140-960]	**0.001**	**0.010**	Ns
**Length of hospital stay (day), median [min-max]**	14 [6-87]	10.5 [6-34]	14 [8-42]	15 [7-87]	**0.007**	**0.049**	Ns
**Readmission at POD90 (%)**	13 (12.3)	3 (25)	6 (28.6)	4 (5.5)	**0.023**	Ns	**0.002**

Abbreviations: *PD* pancreato-duodenectomy; *DP* distal pancreatectomy; *EPI* exocrine pancreatic insufficiency; *POD90* post-operative day 90

### Pathologic findings

The pathological findings are summarized in Table 3. Overall, 33 (34.7%), 52 (54.7%), and 10 patients (10.6%) were classified as grades 1, 2, and 3, respectively. The C-PNET group had significantly lower grades (*P* = 0.002) and higher differentiation grades (*P* = 0.003) when compared with the S-PNET group. The median number of analyzed lymph nodes was higher in both the S-PNET and M-PNET groups than in the C-PNET group (8 and 8 vs 2, respectively, *P* = 0.014). Moreover, the node invasion rate was significantly higher in the S-PNET (40.8%) and M-PNET (23.8%) groups than in the C-PNET group (8.3%, *P* = 0.004). Biopsies were achieved in 96 patients (92.4%). The rates of correlation between the preoperative diagnosis according to initial staging in these patients and the pathological analysis were 89%, 62%, and 33% in the S-PNET, M-PNET, and C-PNET groups, respectively, and these differences were significant (*P* = 0.03).

**Table 3:** Pathological results

**Variables**	**All (*n*** **= 106)**	**C-PNETs (*n*** **= 12)**	**M-PNETs (*n*** **= 21)**	**S-PNETs (*n*** **= 73)**	***P*** **value (C Vs S)**	***P*** **value (C Vs M)**	***P*** **value (M Vs S)**
**Tumor height (mm), median [min-max]**	27 [2-140]	26 [8-60]	30 [6-110]	27.5 [2-140]	Ns	Ns	Ns
**WHO classification**							
Grade 1 (%)	33 (31.1)	9 (75.0)	9 (42.9)	15 (20.5)	**0.002**	Ns	Ns
Grade 2 (%)	52 (49.1)	3 (25.0)	9 (42.9)	40 (54.8)	Ns	Ns	Ns
Grade 3 (%)	10 (9.4)	0	2 (9.5)	8 (11)	Ns	Ns	Ns
Missing data (%)	11 (10.4)	0	1 (4.8)	10 (13.7)	Ns	Ns	Ns
**Ki67/MIB, Median [Min - Max]**	5 [0-95]	2 [1-5]	3.5 [1-90]	5 [0-95]	**0.003**	Ns	Ns
**T status (pTNM)**							
T1 (%)	13 (12.3)	2 (16.7)	2 (9.5)	2 (2.8)	Ns	Ns	Ns
T2 (%)	29 (27.4)	7 (58.3)	9 (42.9)	13 (17.8)	**0.006**	Ns	Ns
T3 (%)	39 (36.8)	1 (8.3)	2 (9.5)	36 (49.3)	Ns	Ns	Ns
T4 (%)	10 (9.4)	0	2 (9.5)	8 (11)	Ns	Ns	Ns
Missing values due to enucleation (%)	15 (14.2)	2 (16.7)	6 (28.6)	7 (9.6)	Ns	Ns	Ns
**Lymph node involvement**							
N0 (%)	38 (35.8)	2 (16.7)	10 (47.6)	26 (35.6)	**0.001**	**0.036**	Ns
N1 (%)	35 (33)	1 (8.3)	5 (23.8)	29 (39.7)	**0.049**	Ns	Ns
NX (%)	33 (31.1)	9 (75)	6 (28.6)	18 (24.7)	**0.001**	**0.014**	Ns
**Lymph nodes analyzed, Median [min-max]**	5 [0-34]	0 [0-11]	6 [0-26]	7 [0-34]	**0.003**	**0.028**	Ns
**R0 resection (%)**	101 (96.2)	12 (100)	21 (100)	68 (93.2)	Ns	Ns	Ns
**Vascular invasion (%)**	21 (19.8)	2 (16.7)	4 (19)	15 (20.5)	Ns	Ns	Ns

Abbreviations: *WHO*, World Health Organization

### Survival

**Fig. 1:**
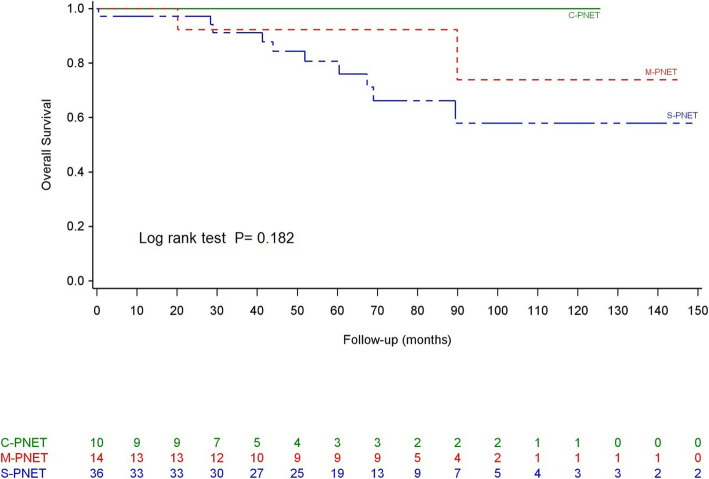
Overall survival after matching

**Fig. 2:**
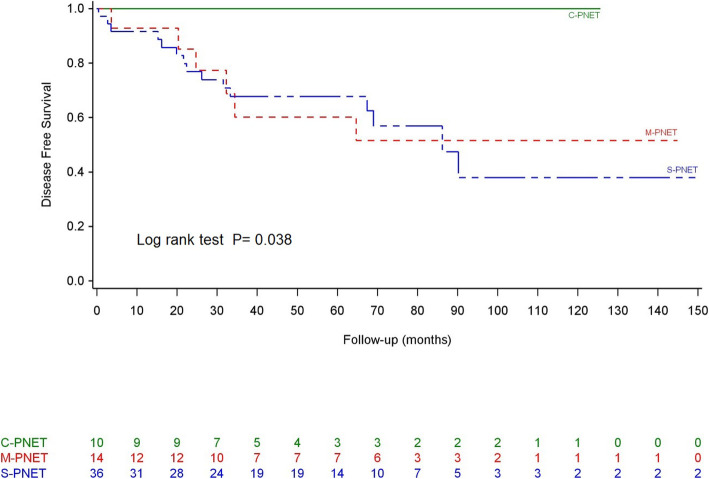
Disease-free survival after matching

No patient was lost to follow up. During a median follow-up of 73.8 months, 38 patients (35.9%) experienced a recurrence, and 24 (22.6%) died from recurrent disease. Interestingly, neither type of event involved patients in the C-PNET group. The OS durations at 1, 3, and 5 years were 96%, 92% and 86%. The median OS durations were comparable (not estimable) in all 3 groups before (*P* = 0.3) and after (*P* = 0.18) matching according to the WHO grade, tumor height, BMI, and age (supplementary Table 5—Fig. 1). However, the median DFS duration was higher in the C-PNET group than in the S-PNET or M-PNET groups after matching (*P* = 0.038; supplementary Table 6—Fig. 2).

## Discussion/conclusion

### Characteristics of the patients, surgeries, and postoperative courses

In this study, we demonstrated that C-PNET (a) was associated with a better prognosis than S- or M-PNET at an equivalent stage and (b) was suspected at preoperative staging in only a third of patients who later received a pathological diagnosis of this tumor type. Our observation that no patient with C-PNET was diagnosed with metastatic disease was consistent with the findings of other series [4, 5]. In contrast, metastatic disease was not uncommon in our other PNET groups, and the neoadjuvant treatment rate was low (7.5%). Not surprisingly, further comparison of the groups according to the tumor localization or surgery type [6] revealed that C-NET was more frequently treated with parenchyma-sparing procedures and less frequently via enlarged resections, and consequently was associated with a shorter operative duration and hospital stay length.

The overall morbidity and mortality rates in our study were similar to those reported previously at high-volume centers [7, 8] and were not influenced by the type of PNET. However, we expected a higher POPF rate than the 37.7% observed in our series, as patients with PNETs often undergo pancreatectomies in a soft pancreatic parenchyma [9]. As the POPF grading was established in 2005 [10], we suppose that our long inclusion period did not enable the identification of grade A POPF, leading to an underestimation of the POPF rate. The postoperative exocrine pancreatic insufficiency rate was more important in S-PNETs, and this was likely attributable to the lower frequency of parenchyma-sparing procedures.

### Histological features

We note that fewer lymph nodes were analyzed in cases involving C-PNETs than in those involving M- or S-PNETs, even after the matching process. This was likely attributable to the observed higher rate of parenchyma-sparing procedures and lower rate of lymph node invasion, as reported previously [11–13]. Staging via imaging and EUS (and eventually associated with biopsies) could efficiently identify a PNET in most patients with only a solid component (correct diagnosis rate: 89%). However, this accuracy decreased to 33% in cases involving pure cystic lesions, thus underscoring the difficulty with affirming a precise diagnosis in patients with unique cystic lesions and the insufficiency of biopsies and/or cyst fluid analyses in this context [12].

### Survival and recurrence

The survival impact of the cystic component remains undetermined because of contradictory results from previous studies [4, 14, 15]. In our series, we observed no statistical difference in OS between the 3 groups, even though the median DFS time was longer in the C-PNET group after matching. However, no patient with C-PNET developed recurrent disease or died during follow-up, whereas these outcomes affected 22.6% of patients in the M- and S-PNET groups. Therefore, the survival curve trends suggested a better prognosis with C-PNET, despite the lack of statistical significance. The lack of an observable significant difference is probably because of the rarity of PNET and the generally good prognosis. A longer follow-up with more events would likely be needed to confirm the apparent superior survival outcomes of patients with C-PNET.

### Perspectives

Our study affirmed that C-PNET was less frequently metastatic at diagnosis, associated with lymphatic invasion, or classified as a high-grade lesion and was potentially associated with better survival outcomes. These findings are consistent with a recent report by the Verona pancreatic group [4]. The existing evidence suggests that whereas the surgical indications of M- or S-PNETs are indisputable, C-PNET could potentially be monitored via follow-up (the “wait and see” policy). Such speculation is quite logical if we assume that in a C-PNET, the tumoral part is represented only by the wall thickness. Therefore, the actual tumoral volume may correspond to a “small” (< 2 cm) S-PNET, for which follow-up is currently recommended [13, 16–18] by the French guidelines [19]. In fact, the major challenge associated with this type of lesion is to confirm the diagnosis of C-PNET, as a misdiagnosis of a more aggressive tumor (e.g., IPMN, mucinous cystadenoma) may lead to unnecessary resection. Accordingly, an indisputable diagnosis at the time of initial staging is essential. In this regard, studies of EUS-guided confocal endomicroscopy [20, 21] and transcriptomic analyses of cystic fluid [22, 23] have been conducted to improve the accuracy of staging and thus spare patients from unnecessary surgery. In our study, due to the long period of inclusion, all patients did not undergo the same imaging as MRI, PET/CT, or Octreotide SPECT/CT. The systematic association of ^68^Ga-DOTATATE PET/CT and ^99m^Tc-Octreotide SPECT/CT to conventional morphologic imaging could be an important help to confirm the diagnosis by their sensitivity and specificity and should be routinely used in the initial staging and the follow-up [24, 25].

Regarding limitations, our study was based on a retrospective design and a long inclusion period. We recognize that our findings may have been impacted by changes in tumor classification and therapeutic strategies during the last 2 decades. However, PNETs are rare tumors. Multi-center studies are often required to analyze a significant sample, and these are associated with biases. Therefore, the single-center setting of our series was a notable strength that enabled homogeneity with respect to preoperative management, surgical procedures, histological analyses, and adjuvant treatments. Moreover, the numbers of C-PNETs and M-PNETs, a relatively important parameter given the rarity of this presentation, were consistent with those reported previously by high-volume pancreatic surgery centers [4, 14].

### Conclusion

C-PNETs are unique entities that appear to have a better prognosis than S- or M-PNETs. Accordingly, a wait-and-see policy, rather than immediate resection, may be prudent in such cases. However, the initial staging of these lesions remains challenging. Further efforts are needed to confirm an accurate diagnosis and spare patients from unnecessary surgery.

## Supplementary information

**Additional file 1: Supplementary Table 4.** Matching process.

**Additional file 2: Supplementary Table 5.** Overall Survival before and after matching.

**Additional file 3: Supplementary Table 6.** Disease Free Survival before and after matching.

## Data Availability

The data that support the findings of this study are available from CHIRPAN No. Sy50955016U but restrictions apply to the availability of these data, which were used under license for the current study, and so are not publicly available. Data are however available from the authors upon reasonable request and with permission of Pr Turrini Olivier and Institut Paoli-Calmettes.

## References

[1] Lykoudis PM, Partelli S, Muffatti F (2019). Treatment challenges in and outside a specialist network setting: pancreatic neuroendocrine tumours. Eur J Surg Oncol.

[2] Metz DC, Jensen RT (2008). Gastrointestinal neuroendocrine tumors: pancreatic endocrine tumors. Gastroenterology..

[3] Modlin IM, Oberg K, Chung DC (2008). Gastroenteropancreatic neuroendocrine tumours. Lancet Oncol.

[4] Paiella S, Marchegiani G, Miotto M (2018). Are cystic pancreatic neuroendocrine tumors an indolent entity results from a single-center surgical series. Neuroendocrinology.

[5] Singhi AD, Chu LC, Tatsas AD (2012). Cystic pancreatic neuroendocrine tumors: a clinicopathologic study. Am J Surg Pathol.

[6] Bordeianou L, Vagefi PA, Sahani D (2008). Cystic pancreatic endocrine neoplasms: a distinct tumor type?. J Am Coll Surg.

[7] Winter JM, Brennan MF, Tang LH (2012). Survival after resection of pancreatic adenocarcinoma: results from a single institution over three decades. Ann Surg Oncol.

[8] Cameron JL, He J (2015). Two thousand consecutive pancreaticoduodenectomies. J Am Coll Surg.

[9] Atema JJ, Jilesen AP, Busch OR, van Gulik TM, Gouma DJ, Nieveen van Dijkum EJ (2015) Pancreatic fistulae after pancreatic resections for neuroendocrine tumours compared with resections for other lesions. HPB (Oxford)17:38–45. 10.1111/hpb.12319 PMC4266439 25041879

[10] Bassi C, Dervenis C, Butturini G (2005). Postoperative pancreatic fistula: an international study group (ISGPF) definition. Surgery.

[11] Beger HG, Siech M, Poch B, Mayer B, Schoenberg MH (2015). Limited surgery for benign tumours of the pancreas: a systematic review. World J Surg.

[12] Duconseil P, Turrini O, Ewald J, et al (2015) ‘Peripheric’ pancreatic cysts: performance of CT scan, MRI and endoscopy according to final pathological examination. HPB (Oxford).17:485–489. 10.1111/hpb.12388 PMC4430777 25691074

[13] Goh BK, Ooi LL, Tan YM (2006). Clinico-pathological features of cystic pancreatic endocrine neoplasms and a comparison with their solid counterparts. Eur J Surg Oncol.

[14] Cloyd JM, Kopecky KE, Norton JA (2016). Neuroendocrine tumors of the pancreas: degree of cystic component predicts prognosis. Surgery.

[15] Gaujoux S, Tang L, Klimstra D (2012). The outcome of resected cystic pancreatic endocrine neoplasms: a case-matched analysis. Surgery.

[16] Lee LC, Grant CS, Salomao DR (2012). Small, nonfunctioning, asymptomatic pancreatic neuroendocrine tumors (PNETs): role for nonoperative management. Surgery.

[17] Rosenberg AM, Friedmann P, Del Rivero J, Libutti SK, Laird AM (2016). Resection versus expectant management of small incidentally discovered nonfunctional pancreatic neuroendocrine tumors. Surgery.

[18] Bettini R, Partelli S, Boninsegna L (2011). Tumor size correlates with malignancy in nonfunctioning pancreatic endocrine tumor. Surgery.

[19] Cadiot G, Baudin E, Couvelard A, Dromain C, Lepage C, Lombard-Bohas C, et al. “Tumeurs Neuroendocrines”. Thésaurus National de Cacnérologie Digestive. Version 2016. Available on www.tncd.org.

[20] Haghighi M, Sethi A, Tavassoly I, Gonda TA, Poneros JM, McBride RB (2019) Diagnosis of pancreatic cystic lesions by virtual slicing: comparison of diagnostic potential of needle-based confocal laser endomicroscopy versus endoscopic ultrasound-guided fine-needle aspiration. J Pathol Inform 10:34. Published 2019 Nov 13. 10.4103/jpi.jpi_32_19 PMC6883479 31799020

[21] Cheesman AR, Zhu H, Liao X (2019). Impact of EUS-guided microforceps biopsy and needle-based confocal laser endomicroscopy on the diagnostic yield and clinical management of pancreatic cystic lesions [published online ahead of print, 2019 Dec 24]. Gastrointest Endosc.

[22] Caglià P, Cannizzaro MT, Tracia A (2015). Cystic pancreatic neuroendocrine tumors: to date a diagnostic challenge. Int J Surg.

[23] Chen L, Nassar A, Kommineni VT (2015). Endoscopic ultrasonography-guided fine-needle aspiration cytology of surgically confirmed cystic pancreatic neuroendocrine tumors: a Mayo Clinic experience. J Am Soc Cytopathol.

[24] Etchebehere EC, de Oliveira SA, Gumz B (2014). 68Ga-DOTATATE PET/CT, 99mTc-HYNIC-octreotide SPECT/CT, and whole-body MR imaging in detection of neuroendocrine tumors: a prospective trial. J Nucl Med..

[25] Fallahi B, Manafi-Farid R, Eftekhari M (2019). Diagnostic efficiency of 68Ga-DOTATATE PET/CT as compared to 99mTc-Octreotide SPECT/CT and conventional morphologic modalities in neuroendocrine tumors. Asia Ocean J Nucl Med Biol..

