# Supplementary material for: A single-center experience with pancreatic cystic neuroendocrine tumors
Source: World J Surg Oncol. 2020 Aug 15;18:208. doi: 10.1186/s12957-020-01994-6 (PMC7429455; doi:10.1186/s12957-020-01994-6)
Supplement: Supplementary file 1 — Additional file 1: Supplementary Table 4. Matching process. [file 12957_2020_1994_MOESM1_ESM.docx]

**Table 4. Population characteristics after matching 1:5 (WHO, Tumor height, BMI, Age)**

| Variables | All  (n=60) | C-PNETs  (n=10) | S + M-PNETs  (n=50) | *p*-value |
| --- | --- | --- | --- | --- |
| **Sexe, Female (%)** | 37 (61.7) | 5 (50) | 32 (64) | ns |
| **Age at surgery > 60 yso (%)** | 26 (43.33) | 3 (30.00) | 23 (46.00) | ns |
| **BMI > 25% (%)** | 30 (50) | 7 (70) | 23 (46) | ns |
| **Tumor height (mm), Median [Min-Max]** | 25 [2-140] | 25 [8-50] | 25 [2-140] | ns |
| **Approach** |  |  |  |  |
| Laparotomy (%) | 50 (83.3) | 7 (70) | 43 (86) | ns |
| Laparoscopy (%) | 8 (13.3) | 3 (30) | 5 (10) | ns |
| Robot-assisted (%) | 2 (3.3) | 0 | 2 (4) | ns |
| **Type of surgery** |  |  |  |  |
| PD (%) | 18 (30) | 1 (10) | 17 (34) | ns |
| DP (%) | 27 (45) | 7 (70) | 20 (40) | ns |
| Isthmic pancreatectomy (%) | 5 (8.3) | 1 (10) | 4 (8) | ns |
| Enucleation (%) | 9 (15) | 1 (10) | 8 (16) | ns |
| Completion of pancreatectomy (%) | 2 (3.3) | 0 | 2 | ns |
| Enlarged resection (%) | 18 (30) | 0 | 18 (36) | **0.023** |
| Parenchyma sparing (%) | 13 (21.7) | 2 (20) | 11 (22) | ns |
| **WHO Classification** |  |  |  |  |
| Grade 1 (%) | 36 (60) | 9 (90) | 27 (54) | ns |
| Grade 2 (%) | 22 (36.7) | 1 (10) | 21 (42) | ns |
| Grade 3 (%) | 2 (3.3) | 0 | 2 (4) | ns |
| **Lymph node involvement** |  |  |  |  |
| N0 (%) | 23 (38.3) | 2 (20) | 21 (42) | **0.026** |
| N1 (%) | 17 (28.3) | 1 (10) | 16 (32) | ns |
| NX (%) | 20 (33.3) | 7 (70) | 13 (26) | **0.011** |
| **Lymph nodes analysed, Median [Min-Max]** | 7.00 [0.00-26.00] | 0.00 [0.00-11.00] | 8.00 [0.00-26.00] | **0.015** |
| **R0 resection (%)** | 59 (98.3) | 10 (100) | 49 (98) | ns |
| **Vascular invasion (%)** | 10 (16.7) | 1 (10) | 9 (18) | ns |
| **Events** |  |  |  |  |
| Recurrence (%) | 18 (30) | 0 | 18 (36) | **0.023** |
| Death (%) | 12 (20) | 0 | 12 (24) | ns |
| Specific Death (%) | 11 (18.3) | 0 | 11 (22) | ns |

*Abbreviations: BMI: Body Mass Index; PD: Pancreato-Duodenectomy; DP: Distal Pancreatectomy; WHO: World Health Organization*
