# Supplementary material for: A single-center experience with pancreatic cystic neuroendocrine tumors
Source: World J Surg Oncol. 2020 Aug 15;18:208. doi: 10.1186/s12957-020-01994-6 (PMC7429455; doi:10.1186/s12957-020-01994-6)
Supplement: Supplementary file 2 — Additional file 2: Supplementary Table 5. Overall Survival before and after matching. [file 12957_2020_1994_MOESM2_ESM.docx]

**Table 5a. Overall Survival before matching**

| Groups | Number of observations | Number of events (%) | Median [95% CI] | 12 M | 36M | 60 M | *P value* |
| --- | --- | --- | --- | --- | --- | --- | --- |
| **C-PNETs** | 12 | 0 (0.00%) | . | 1.00 [1.00,1.00] | 1.00 [1.00,1.00] | 1.00 [1.00,1.00] | 0.303 |
| **M-PNETs** | 21 | 4 (19.05%) | . | 0.95 [0.71,0.99] | 0.90 [0.65,0.97] | 0.90 [0.65,0.97] |  |
| S-PNETs | 73 | 22 (30.14%) | 160.00[92.79,.] | 0.96 [0.88,0.99] | 0.91 [0.82,0.96] | 0.83 [0.71,0.90] |  |

**Table 5b. Overall Survival after matching**

| Groups | Number of observations | Number of events (%) | Median [95% CI] | 12 M | 36M | 60 M | *P value* |
| --- | --- | --- | --- | --- | --- | --- | --- |
| **C-PNETs** | 10 | 0 (0.00%) | . | 1.00 [1.00,1.00] | 1.00 [1.00,1.00] | 1.00 [1.00,1.00] | 0.182 |
| M + S-PNETs | 50 | 12 (24.00%) | . | 0.98 [0.87,1.00] | 0.92 [0.79,0.97] | 0.84 [0.69,0.92] |  |
