# Supplementary material for: A single-center experience with pancreatic cystic neuroendocrine tumors
Source: World J Surg Oncol. 2020 Aug 15;18:208. doi: 10.1186/s12957-020-01994-6 (PMC7429455; doi:10.1186/s12957-020-01994-6)
Supplement: Supplementary file 3 — Additional file 3: Supplementary Table 6. Disease Free Survival before and after matching. [file 12957_2020_1994_MOESM3_ESM.docx]

**Table 6a. Disease Free Survival before matching**

| Groups | Number of observations | Number of events (%) | Median [95% CI] | 12 M | 36M | 60 M | *P value* |
| --- | --- | --- | --- | --- | --- | --- | --- |
| **C-PNETs** | 12 | 0 (0.00%) | . | 1.00 [1.00,1.00] | 1.00 [1.00,1.00] | 1.00 [1.00,1.00] | 0.058 |
| **M-PNETs** | 21 | 9 (42.86%) | . | 0.86 [0.62,0.95] | 0.58 [0.33,0.77] | 0.58 [0.33,0.77] |  |
| S-PNETs | 73 | 35 (47.95%) | 80.89[52.75,107.93] | 0.86 [0.76,0.92] | 0.64 [0.52,0.74] | 0.62 [0.50,0.73] |  |

**Table 6b. Disease Free Survival after matching**

| Groups | Number of observations | Number of events (%) | Median [95% CI] | 12 M | 36M | 60 M | *P value* |
| --- | --- | --- | --- | --- | --- | --- | --- |
| **C-PNETs** | 10 | 0 (0.00%) | . | 1.00 [1.00,1.00] | 1.00 [1.00,1.00] | 1.00 [1.00,1.00] | **0.038** |
| M + S-PNETs | 50 | 21 (42.00%) | 86.16[64.72,.] | 0.92 [0.80,0.97] | 0.66 [0.50,0.78] | 0.66 [0.50,0.78] |  |
